# Distributions of Wall Heat Flux and Wall Shear Stress and their Interrelation During Head-on Quenching of Premixed Flames within Turbulent Boundary Layers

**DOI:** 10.1007/s10494-024-00633-4

**Published:** 2025-01-09

**Authors:** Vishnu Mohan, Umair Ahmed, Nilanjan Chakraborty

**Affiliations:** School of Engineering, Newcastle University, Newcastle-upon-Tyne, Tyne and Wear NE1 7RU United Kingdom

**Keywords:** Flame-wall interaction, Head-on quenching, Turbulent boundary layer, Wall heat flux, Wall shear stress, Proper orthogonal decomposition (POD), Direct numerical simulations (DNS)

## Abstract

The statistical behaviours of wall heat flux and wall shear stress and their interdependence during unsteady head-on quenching of statistically planar turbulent premixed flames within turbulent boundary layers due to heat loss through the cold wall have been analysed using three-dimensional Direct Numerical Simulation data with friction Reynolds numbers of $Re_\tau =110$ and 180. In both cases, the mean wall shear stress decreases during flame-wall interaction, whereas the mean wall heat flux magnitude increases with time as the flame approaches the wall and eventually assumes a maximum value before decreasing with the progress of flame quenching. The integral length scales of wall heat flux in both streamwise and spanwise directions have been found to grow with time after the maximum mean heat flux magnitude is obtained for the two $Re_\tau$ cases considered. However, the integral length scale of wall shear stress in the streamwise direction grows but the integral length scale of wall shear stress in the spanwise direction decreases with time after the maximum mean heat flux magnitude is reached. Moreover, the correlation coefficient between the wall heat flux magnitude and wall shear stress becomes increasingly negative while the mean wall heat flux increases with time, but this negative correlation weakens with the progress of flame quenching. The first few (i.e., most energetic) Proper Orthogonal Decomposition (POD) modes of wall shear stress and the wall heat flux magnitude have been found to capture the qualitative nature of the correlation between these quantities and their spatial variations. It is found that tens of most energetic POD modes are needed to capture the mean and variances of wall heat flux and wall shear stress. The number of most energetic modes, which contribute significantly to the statistics of both wall heat flux and wall shear stress, decreases with decreasing $Re_\tau$ and also with the progress of flame quenching due to the weakening of turbulence effects.

## Introduction

The development of fuel-efficient engine technologies across all transportation sectors in response to the need for environment-friendly electric powertrains beyond 2035 (International Energy Agency, 2015) gives rise to downsized combustors with high power density. The surface-to-volume ratio of the combustor increases with the downsizing, which leads to high probabilities of flame quenching in turbulent boundary layers with high wall heat flux magnitudes. The wall heat flux during flame-wall interaction (FWI) within turbulent boundary layers determines the design of cooling processes for safe operation and overall durability of the combustors (Poinsot and Veynante 2001). The advancements in experimental diagnostics and high-performance computing have given rise to several important analyses on wall heat flux statistics during FWI (Vosen et al. 1985; Huang et al. 1988; Poinsot et al. 1993; Gruber et al. 2012; Lai and Chakraborty 2016; Jainski et al. 2018; Zhao et al. 2018; Ghai et al. 2022; Lai et al. 2022; Ghai et al. 2023; Zhao et al. 2022; Bruneaux et al. 1996; Jarosinski 1986; Alshaalan and Rutland 2002; Gruber et al. 2010; Lai et al. 2019; Konstantinou et al. 2021; Lai et al. 2018; Rißmann et al. 2017; Jainski et al. 2017). The maximum wall heat flux magnitude and the non-dimensional flame quenching distance (i.e., henceforth will be referred to as the Peclet number) have been analysed for conventional laminar head-on quenching (HOQ) of premixed turbulent flames by isothermal inert walls using experimental means (Vosen et al. 1985; Huang et al. 1988; Poinsot et al. 1993). These experimental findings have been validated by 2D simple chemistry Direct Numerical Simulation (DNS) (Jarosinski 1986) and 3D detailed chemistry DNS (Lai and Chakraborty 2016; Lai et al. 2018, 2022) data. Zhao et al. (2018) proposed a semi-analytical model for wall heat flux during flame quenching in a configuration where the flame impinges on an isothermal cold surface based on simple chemistry DNS data. This was subsequently validated by detailed chemistry DNS data (Zhao et al. 2022). The analyses of wall heat transfer statistics within turbulent boundary layers were pioneered by Alshaalan and Rutland (2002) for oblique wall-quenching (OWQ) of turbulent V-flames with isothermal inert walls, which revealed the role of flame-vortex interaction on wall heat transfer using 3D DNS data. Lai et al. (2019) analysed the effects of turbulent flow topology on wall heat flux using DNS data of OWQ of turbulent V-flames by isothermal inert walls. Ghai et al. (2022) used DNS data of statistically stationary OWQ of V-flames to demonstrate that the turbulent burning velocity is closely related to wall heat flux magnitude during statistically stationary FWI within turbulent boundary layers. Recently, the wall heat transfer statistics during OWQ within turbulent boundary layers for different fuel Lewis numbers have been analysed by Ghai et al. (2023) using DNS data. The analysis by Ghai et al. (2023) indicated that the (a) orientation of the flame normal vector with the wall normal vector, (b) flame curvature distribution in the near-wall region, (c) wall shear stress distribution and (d) coherent flow structures can potentially affect the wall heat flux distribution during FWI. However, the underlying turbulent structure of wall heat flux magnitude distribution and its correlation with the distributions of wall shear stress during premixed FWI within turbulent boundary layers are yet to be analysed in detail. The present analysis addresses this aspect by considering DNS data of HOQ of statistically planar turbulent premixed flame approaching an isothermal inert wall across turbulent boundary layers for two different friction Reynolds numbers $Re_\tau =110$ and 180. In this configuration, the flame quenches when it reaches close to the wall due to wall heat loss. The wall heat flux magnitude is affected by the several aforementioned factors indicated by Ghai et al. (2023) and thus complex heat flux structures are expected at the wall.

The present work analyses wall heat flux and wall shear stress distributions and their correlation during HOQ using Proper Orthogonal Decomposition (POD). Note that POD is often employed as a tool to identify the dominant features in the underlying turbulent flow field. Rönnberg and Duwig (2021) utilised extended POD to identify coherent structures present in non-reacting impinging jets which were responsible for heat transfer to the wall. It is claimed that among all possible linear decomposition methods, for a given number of modes, POD provides projections on the subspace which contains the most amount of energy (Berkooz et al. 1993). When POD is applied to the velocity field in turbulent flows it provides the dominant structures with the energy content decreasing with increasing modes, whereas POD of other scalars or vectors provide the modes in decreasing order of variance (Berkooz et al. 1993). Turbulent flows are known to have a broad range of spatio-temporal scales making them difficult to analyse and simulate. This challenge is exacerbated further due to additional length and time scales associated with chemical reactions and molecular diffusion rates during FWI. Thus, the distributions of wall shear stress and wall heat flux magnitude are expected to show high levels of complexity. In Large Eddy Simulations (LES), small-scale physics is modelled using sub-grid closures. The data generated for wall heat flux and wall shear stress from LES would mimic the data generated by the first few POD modes of the DNS data, as POD modes for homogeneous data are equivalent to the Fourier modes of the two-point correlation function of the data in question (Berkooz et al. 1993). Thus, the dominant structures of wall shear stress and wall heat flux during turbulent premixed FWI and their correlation will be analysed using POD in Sect. 3 of this paper. The information on the DNS database is provided in the next section of this paper.

## DNS Database

A three-dimensional DNS database of HOQ for statistically planar turbulent premixed flames due to the interaction with inert isothermal walls has been considered for the current analysis. The chemical mechanism for this database is simplified by a single-step Arrhenius-type chemical reaction (unit mass of Fuel + *s* unit mass of Oxidiser $\rightarrow$ (1+*s*) unit mass of Products, where *s* is the stoichiometric oxidiser-fuel mass ratio) in the interest of computational economy for the current analysis. The value of stoichiometric oxygen to fuel ratio by mass *s* is taken to be 4.0 which is representative of stoichiometric methane-air premixed flames. The unburned gas temperature $T_R$ is taken to be 730*K*, which yields a Zel’dovich parameter, $\beta =T_a (T_{ad}-T_R)/T_{ad}^2$ of 6.0 (where $T_a,T_{ad}$ and $T_R$ is the activation, adiabatic and reactant temperatures, respectively), and a heat release rate parameter of $\tau =(T_{ad}-T_R)/T_R =2.3$. The Lewis number of all the species is taken to be unity and standard values are considered for the Prandtl number *Pr* and the ratio of specific heat capacities, $\gamma$ (i.e., $Pr=0.7$, $\gamma =1.4$). It has been demonstrated in previous analyses (Lai et al. 2018, 2022; Zhao et al. 2022) that the statistical behaviours of heat flux magnitude, flame quenching distance and wall-normal temperature variation for hydrocarbon-air flames are not affected by the simplification of chemistry and thus, it is expected that the conclusions of this analysis will at least be valid in a qualitative sense. The single-step chemistry used in the work represents methane-air combustion in which recombination-type reactions are less intense than hydrogen-air combustion (Lai et al. 2022). A unity Lewis number condition is considered for the current analysis, which is a reasonable assumption for stoichiometric methane-air flames. A recent analysis (Ghai et al. 2023) indicated that the qualitative nature of wall shear stress and wall heat flux distributions do not change (but there are quantitative differences) for effective fuel Lewis numbers ranging from 0.6 to 1.4. It was demonstrated in several previous analyses that the heat flux statistics obtained from detailed chemistry DNS for methane-air (Lai et al. 2018, 2022) and hydrogen-air (Gruber et al. 2010; Lai et al. 2022; Zhao et al. 2023) flames are qualitatively (and mostly quantitatively) similar to unity Lewis number simple chemistry DNS data (Alshaalan and Rutland 2002; Lai and Chakraborty 2016; Zhao et al. 2018).

The simulations used for the current analysis are performed using a three-dimensional code called SENGA+ (Jenkins and Cant 1999) where the first and second-order spatial derivatives are calculated using a 10^th^-order finite difference central scheme for the internal grid points, but the order of accuracy gradually reduces to a one-sided 2^nd^ order scheme for the non-periodic boundaries. An explicit 3^rd^-order Runge–Kutta scheme is used for time advancement. The present analysis considers a configuration where a turbulent boundary layer is formed on top of a chemically inert wall and the initial flow conditions for the reacting flow simulations have been generated using fully developed non-reacting turbulent channel flow solutions corresponding to $Re_\tau =(\rho _R u_{\tau ,NR} h)/\mu _R =110$ and 180 where $u_{\tau ,NR}=\sqrt{(|\tau _{w,NR} |/\rho }$ and $\tau _{w,NR}$ are the friction velocity and wall shear stress for the fully developed non-reacting channel flow, respectively, $\rho _R$ is the unburned gas density, $\mu _R$ is the unburned gas viscosity and *h* is the channel half height corresponding to the non-reacting fully developed channel flow solution. The friction Reynolds numbers considered here are comparable to the recent FWI experiments (Zentgraf et al. 2024). The simulation domain is taken to be $10.69h\times 1.33h\times 4h$, which is discretised by equidistant cartesian grids of $1920\times 240\times 720$ and $3200\times 400\times 1200$ for $Re_\tau =110$ and 180, respectively. These grids ensure that $y^+_{NR}=(\rho _R u_{\tau ,NR} y)/\mu _R$ for the wall adjacent grid points are smaller than 0.6 and the thermal flame thickness $\delta _{th}=(T_{ad}-T_R)/max|\nabla T|_L$ is resolved using at least 8 grid points. The non-reacting flow simulations have been benchmarked with respect to previously reported results available in the literature and an excellent agreement was obtained. Interested readers are referred to (Ahmed et al. 2021, 2023) for further information in this regard.

A periodic flow configuration is utilised for the simulations where periodic boundary conditions are adopted for both streamwise (i.e. *x*-direction) and spanwise (i.e. *z*- direction) directions and a pressure gradient (i.e., $-\partial p/\partial x=\rho u_{\tau ,NR}^2/h$, where *p* is the pressure) is imposed in the streamwise direction (Ahmed et al. 2021). The wall at $y=0$ is taken to be no-slip impenetrable, inert (i.e., velocity components in wall normal and tangential directions are zero and mass flux in the wall-normal direction vanishes), and an isothermal thermal wall boundary condition (i.e., $T_w=T_R$) is imposed at the wall. The boundary at $y/h=1.33$ is taken to be partially non-reflecting following an improved Navier–Stokes Characteristic Boundary Conditions (NSCBC) technique (Yoo and Im 2007). For both $Re_\tau =110$ and 180 cases, the steady 1-D laminar flame simulation is used to initialise the reacting scalar field in such a manner that the reaction progress variable $c=(Y_{FR}-Y_F)/(Y_{FR}-Y_{FP})=0.5$ (where the subscripts *R* and *P* represent the fresh reactant and fully burned products, respectively) isosurface is located at $y/h \approx 0.85$ with the reactant side facing the wall. The ratio of the laminar burning velocity $S_L$ to non-reacting flow friction velocity $u_{\tau ,NR}$ remains 0.7 for both cases and the simulations are conducted for a maximum of 2.0 flow through time based on the maximum axial mean velocity, and this time is equivalent to $21.30t_f$ and $30.30t_f$ for $Re_\tau =110$ and 180, respectively, with $t_f=\delta _{th}/S_L$ being the chemical time scale. In the simulations the flame propagates towards the wall and eventually quenches due to heat loss through the wall for both cases considered here, but the boundary layer without any flame-wall interaction (keeping everything else the same) does not show any significant changes in the flow statistics for the simulation times considered (Ahmed et al. 2021, 2023).

## Results and Discussions

**Fig. 1:**
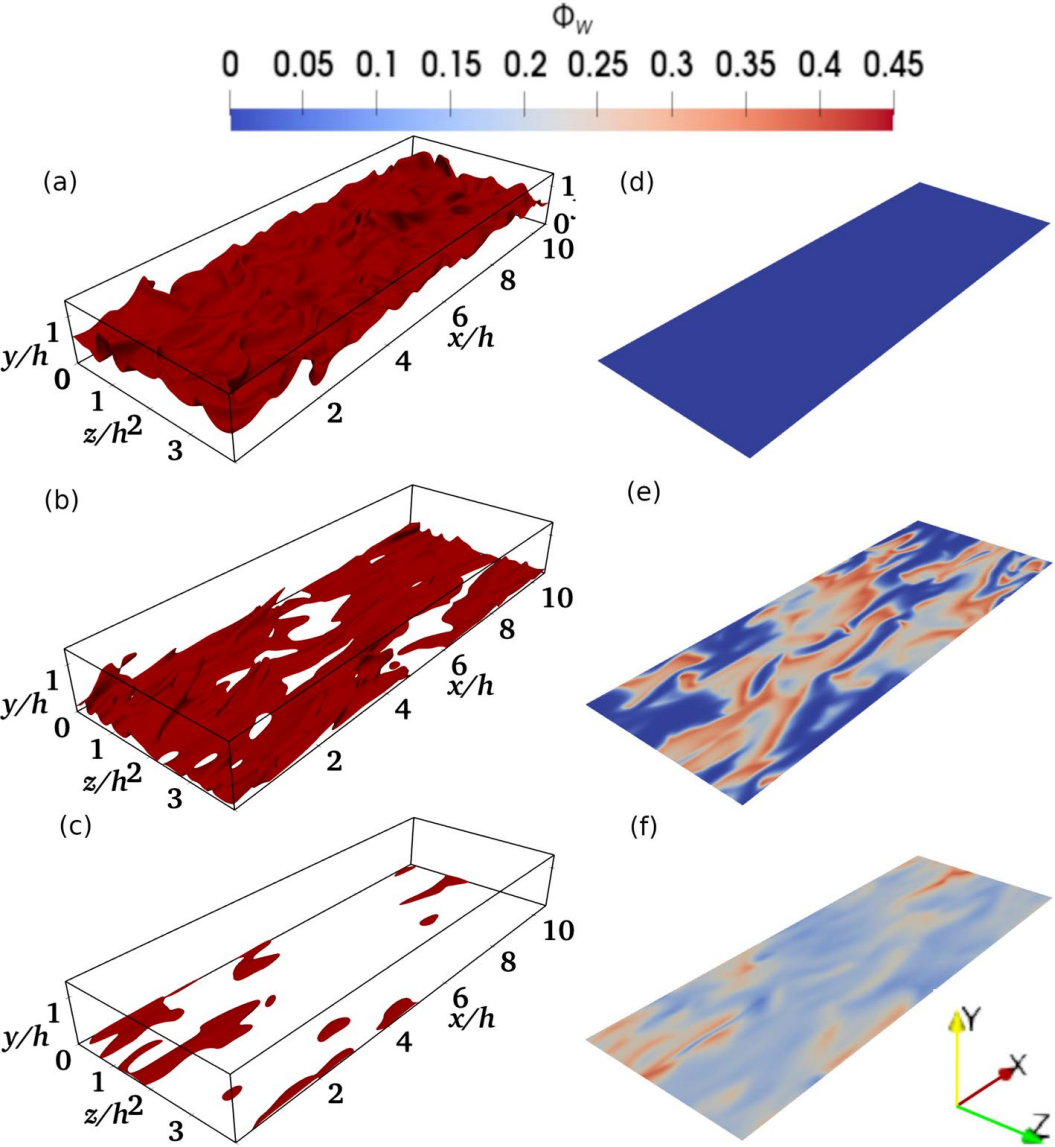
The instantaneous distributions of $c=0.8$ isosurface (**a**–**c**) and the normalised wall heat flux magnitude $\Phi _w=|q_w|/[\rho _Rc_{pR}S_L(T_{ad}-T_R)]$ (**d**–**f**) at $t/t_f=$ (a,d) 3.99, (b,e) 13.12 and (c,f) 16.27 for $Re_\tau =110$

The instantaneous distributions of $c=0.8$ isosurface and the normalised heat flux magnitude $\Phi _w=|q_w|/[\rho _Rc_{pR}S_L(T_{ad}-T_R)]$ on the wall at $t/t_f=3.99, t/t_f=13.12$ and $t/t_f =16.27$ for the $Re_\tau =110$ case are shown in Fig. 1 where $q_w=-\lambda (\partial T/\partial y)_w$ is the wall heat flux with $c_{pR}$ and $\lambda$ being the specific heat at constant pressure of the unburned gas mixture and thermal conductivity, respectively and the subscript ’*w*’ is used to refer to wall quantities. In the case of the simplified thermochemistry employed in this work, the maximum reaction rate of the reaction progress variable for the unstretched laminar premixed flame occurs at $c \approx 0.8$ and thus the $c=0.8$ isosurface can be considered to be the flame surface. The time instants shown in Fig. 1 (in the increasing order) correspond to the normalised wall-normal distances of $y/h=0.72,0.06$ and 0.03 of non-dimensional Favre-averaged temperature $\tilde{\theta } = (\tilde{T}-T_R)/(T_{ad}-T_R)=0.5$, respectively, where the Reynolds and Favre averages (i.e., $\bar{q}$ and $\tilde{q}=\bar{\rho q}/\bar{\rho }$ for a general quantity *q*) are evaluated by ensemble averaging the quantities over homogeneous (i.e., *x* and *z*) directions for a fixed value of *y* at a given instant of time. It is worth noting that the time instants corresponding to the normalised wall-normal distances of $y/h=0.72,0.06$ and 0.03 will be used as exemplars in subsequent figures for both $Re_\tau =110$ and 180. These $t/t_f$ values, in increasing order, are representative of the time instants when the flame remains sufficiently away from the wall, initiation of quenching as a result of FWI and the advanced stage of flame quenching, respectively.

**Fig. 2:**
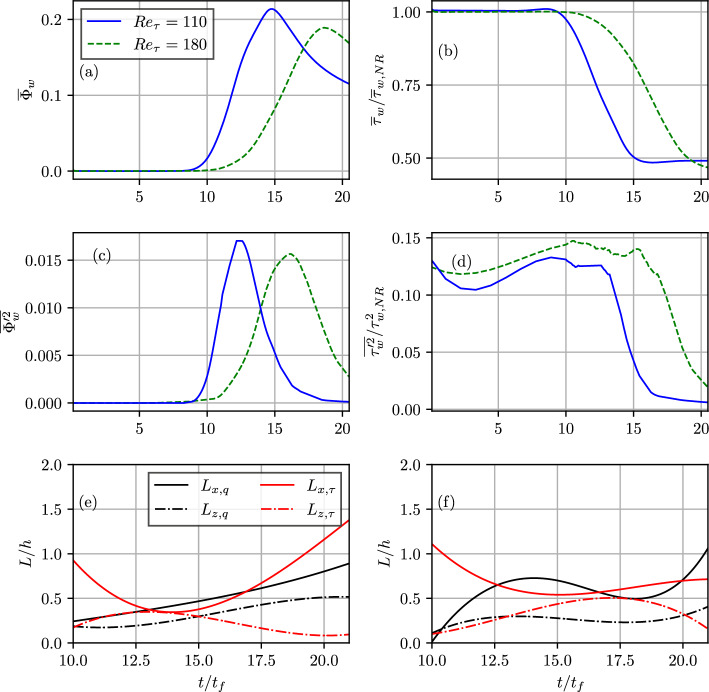
Temporal evolutions of **a** normalised mean heat flux magnitude $\bar{\Phi }_w=|\bar{q}_w|/[\rho _Rc_{pu}S_L(T_{ad}-T_R)]$, **b** normalised mean wall shear stress $\bar{\tau }_w/\tau _{w,NR}$, **c** variance of normalised heat flux magnitude $\overline{\Phi _w'^2}$, **d** variance of normalised wall shear stress magnitude $\overline{\tau _w'^2}/\overline{\tau }_{w,NR}^2$, and normalised streamwise and spanwise integral length scales of wall heat flux (i.e., $L_{x,q}/h$ and $L_{z,q}/h$) and wall shear stress ($L_{x,\tau }/h$ and $L_{z,\tau }/h$) for **e** $Re_\tau =110$ and **f** $Re_\tau =180$ cases

It can be seen from Fig. 1 that the flame propagates towards the wall as time progresses and eventually quenches (indicated by broken islands of $c=0.8$ instead of a continuous isosurface) due to wall heat loss in the vicinity of the wall. It can further be seen from Fig. 1 that $\Phi _w$ is zero when the flame is away from the wall but distributions of significant non-zero values of $\Phi _w$ are obtained when the flame starts to interact with the wall. However, $\Phi _w$ values start to decrease after flame quenching with the thickening of the thermal boundary layer. This can further be substantiated by the temporal evolutions of the normalised magnitude of the mean wall heat flux $\bar{\Phi }_w=|\bar{q}_w|/[\rho _Rc_{pR}S_L(T_{ad}-T_R)]$ in Fig. 2a, which shows $\bar{\Phi }_w$ remains zero before the initiation of FWI and it obtains a maximum value during HOQ before decreasing again during the final stage of quenching. The corresponding temporal evolutions of normalised mean wall shear stress $\bar{\tau }_w/\tau _{w,NR}$ (where $\tau _w=\mu (\partial u/\partial y)_w$ is the wall shear stress and the subscript ‘*NR*’ refers to non-reacting flow values with unburned gas properties) are shown in Fig. 2b, which shows that $\bar{\tau }_w/\tau _{w,NR}$ starts to decrease from 1.0 when $\bar{\Phi }_w$ assumes non-zero values (see Fig. 2a) and eventually settles to a value after the maximum value of $\bar{\Phi }_w$ is reached and $\bar{\Phi }_w$ starts to decrease with time. In the HOQ configuration, the mean flame normal acceleration takes place in the wall-normal direction due to thermal expansion induced by heat release, which gives rise to a redistribution of momentum from the wall tangential direction to the wall-normal direction because the changes in wall-normal velocity component also modify the wall-tangential velocity through the mass conservation equation. This mechanism acts to reduce the magnitude of $(\partial u/\partial y)_w$ during FWI (Ahmed et al. 2023). Interested readers are referred to Ahmed et al. (2023) for further discussion on this aspect, which is not repeated here for the sake of brevity. The variances of the wall heat flux, $\overline{{\Phi ^{'}}_w^2}$, and wall shear stress, $\overline{{\tau ^{'}}_w^2}/\overline{\tau }_{w,NR}^2$ are shown in Figs. 2c and d, respectively. The wall shear stress variance increases as the flame approaches the wall and decreases when non-zero wall heat flux is observed. The increase in wall shear stress variance is due to the turbulent structures in the boundary layer being affected by the presence of the flame. The variance of wall shear stress decreases as the mean wall shear stress decreases due to the redistribution of momentum from the streamwise direction to the wall normal direction with the progress of FWI. The variance of the wall heat flux increases as FWI is initiated and begins to decrease with the progress of flame quenching. The variance of wall heat flux and shear stress are seen to be zero at later times even when the corresponding mean values are non-zero. This implies that the flow tends to become laminar at the advanced stage of flame quenching.

The temporal evolution of the normalised integral length scale in streamwise and spanwise directions for both wall heat flux (i.e., $L_{x,q}$ and $L_{z,q}$) and wall shear stress ($L_{x,\tau }$ and $L_{z,\tau }$) are shown in Figs. 2e and f, where the length scales at a given instant of time are evaluated based on two-point correlations of a quantity $\theta$ (which can be either $\Phi _w$ or $\tau _w$) in the following manner:

1 $$\begin{aligned} L_{x,\theta }= & \int _0^\infty \frac{\langle \theta '(x,z,t)\theta '(x+r,z,t)\rangle }{\langle \theta '(x,z,t)\theta '(x,z,t)\rangle } dr \end{aligned}$$

2 $$\begin{aligned} L_{z,\theta }= & \int _0^\infty \frac{\langle \theta '(x,z,t)\theta '(x,z+r,t)\rangle }{\langle \theta '(x,z,t)\theta '(x,z,t)\rangle } dr \end{aligned}$$

where $\langle \cdots \rangle$ is the ensemble-averaged value of $\theta$ in the statistically homogeneous direction and $\theta '=\theta (x,z,t)-\langle \theta \rangle$ is the local fluctuation of the quantity in question. It can be seen from Figs. 2e and f that length scales in the streamwise direction (i.e., $L_{x,q}$ and $L_{x,\tau }$) assume higher values than those in the spanwise direction (i.e., $L_{z,q}$ and $L_{z,\tau }$) at all stages of FWI. A comparison between Figs. 2e-f reveals that the temporal variation of the integral length scale associated with wall heat flux in general increases with $t/t_f$. By contrast, the longitudinal length scale associated with wall shear stress decreases from its non-reacting value with time before attaining a minimum value but an increasing trend with time is observed after the peak $\bar{\Phi }_w$ is obtained. The spanwise integral length scale of wall shear stress increases with time and attains a maximum value before exhibiting a decreasing trend with time at the final stages of flame quenching. At the onset of FWI, sporadic spots of high heat flux magnitude appear on the wall surface. The length scale associated with the wall heat flux increases as FWI progresses. The regions of high wall heat flux are associated with high thermal expansion, which in turn redistributes the streamwise momentum in the wall normal direction affecting the wall shear stress (Ahmed et al. 2023). Thus, the length scale associated with $\tau _w$ decreases from its non-reacting value and attains a value that is similar to the wall heat flux. During FWI, both $\tau _w$ and $\Phi _w$ maintain similar lengthscale until the maximum $\bar{\Phi }_w$ is reached. Thereafter, the flame quenches leading to the decay in $\Phi _w$ fluctuations, which is apparent from the increase in the longitudinal and spanwise length scale of the wall heat flux. On the other hand, post-quenching, the longitudinal (spanwise) integral length scale of wall shear stress increases (decreases) to approach the corresponding non-reacting shear stress length scales.

**Fig. 3:**
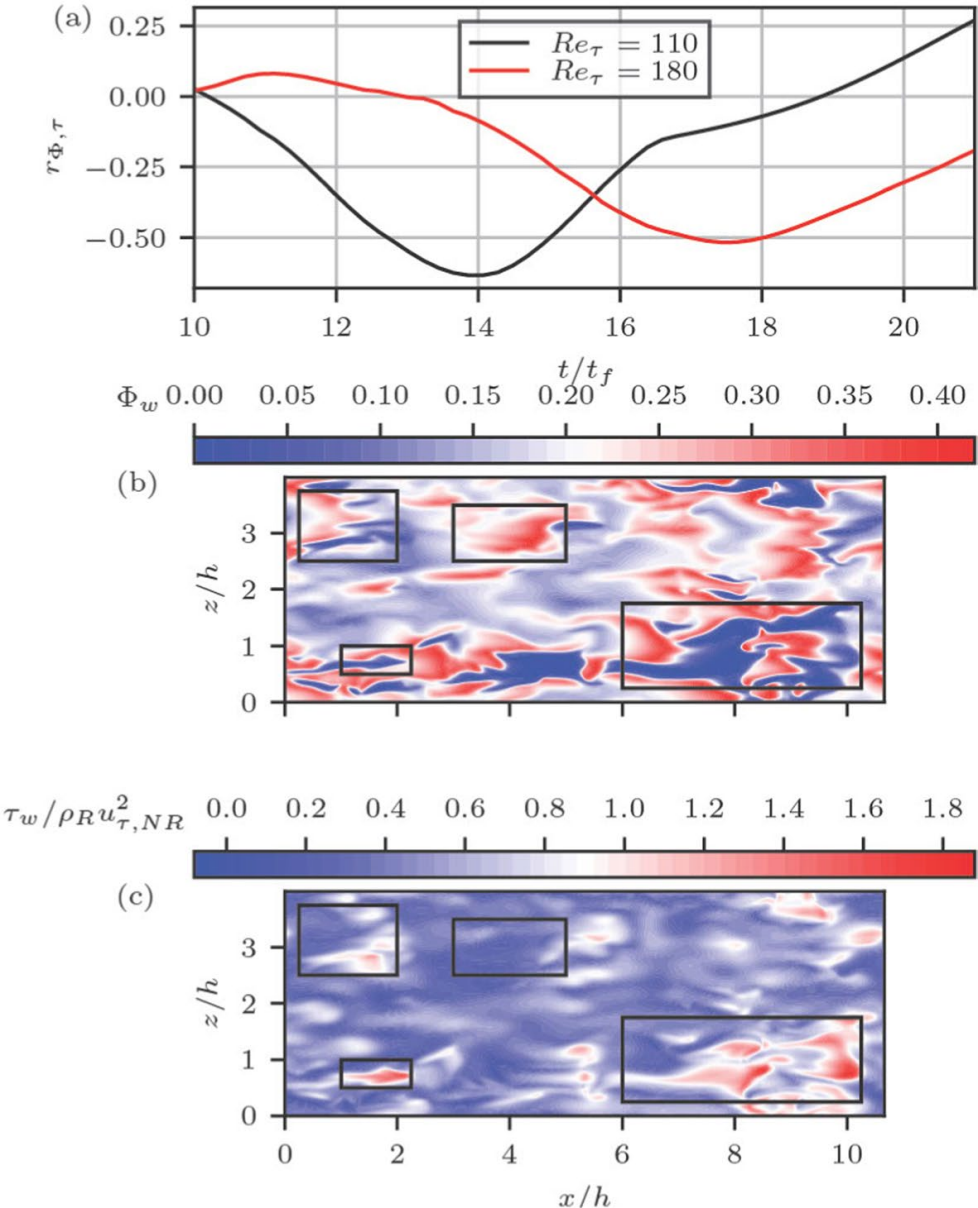
**a** Temporal evolution of the correlation coefficient *r*(*t*) between $\Phi _w$ and $\tau _w$; spatial distributions of **b** $\Phi _w$ and **c** $\tau _w/\rho _R u_{\tau ,NR}^2$ for the $Re_\tau =180$ case at the time instant for which the maximum value of $\bar{{\Phi }}_w$ is obtained

It has already been discussed that heat flux and shear stress become interrelated during FWI. Thus, it is worthwhile to consider the correlation coefficient between the wall heat flux and wall shear stress $r_{\Phi \tau }(t)$, which is defined as:

3 $$\begin{aligned} r_{\Phi \tau }(t)=\frac{\langle (\Phi _w-\bar{\Phi }_w)(\tau _w-\bar{\tau }_w)\rangle }{\sqrt{\langle (\Phi _w-\bar{\Phi }_w)^2\rangle \times \langle (\tau _w-\bar{\tau }_w)^2}\rangle }. \end{aligned}$$

The temporal evolution of $r_{\Phi \tau }(t)$ is shown in Fig. 3a for both $Re_\tau =110$ and 180 cases, which upon comparison with Fig. 2a reveals that ${\Phi }_w$ and $\tau _w$ remain uncorrelated before the initiation of FWI and the correlation coefficient becomes increasingly negative with the progress of HOQ and assumes the maximum magnitude at a value of $t/t_f$ for which the maximum value of $\bar{\Phi }_w$ is obtained, and subsequently, the negative correlation strength weakens with time. The spatial distributions of $\Phi _w$ and $\tau _w/\rho _R u_{\tau ,NR}^2$ for the $Re_\tau =180$ case at the time instant for which the maximum value of $\bar{\Phi }_w$ is obtained are shown in Figs. 3b and c, respectively. A comparison between Figs. 3b and c reveals that the islands of high values of $\Phi _w$ are associated with small values of $\tau _w$ and *vice versa*, and a similar behaviour is obtained for the $Re_\tau =110$ case at the time corresponding to the maximum value of $\Phi _w$. Some of the regions showing opposing trends in $\Phi _w$ and $\tau _w$ are marked with black boxes in Figs. 3b and c. The locations of the high wall heat flux prior to flame quenching are also associated with high wall-normal density gradients (because the thermodynamic pressure does not change in this configuration), which gives rise to an augmentation of the wall-normal velocity gradient due to thermal expansion at the expense of the wall-normal gradient of the streamwise velocity component (Ahmed et al. 2021, 2023). This mechanism strengthens as the flame approaches the wall and thus the correlation between $\Phi _w$ and $\tau _w$ strengthens with time until the maximum value of $\bar{\Phi }_w$ is obtained when the flame can be considered to be the closest to the wall in the mean sense. Upon quenching, the thermal gradient decreases and the effects of thermal expansion weaken with time so the reduction in wall-normal thermal gradient due to thermal diffusion does not have any major influence on the wall-normal gradient of the streamwise velocity component. This is reflected in the weak correlation between $\Phi _w$ and $\tau _w$ at the advanced stages of flame quenching.

**Fig. 4:**
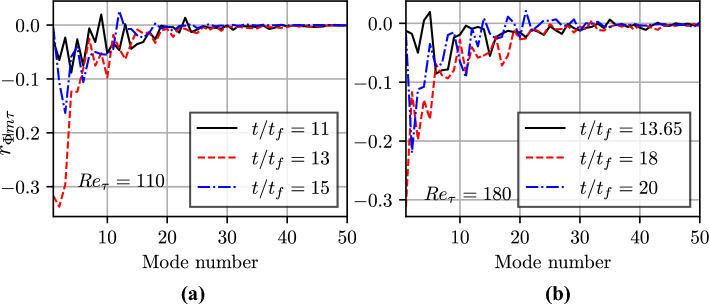
Temporal evolutions of the correlation coefficient of different POD modes of wall heat flux with wall shear stress during the HOQ for $Re_\tau =$ **a** 110 and **b** 180

It has already been demonstrated in Fig. 2 that the integral length scales of wall heat flux are similar to those of wall shear stress and these length scales vary as FWI progresses. To explain this behaviour, POD is employed in the current study. Note that POD is a procedure for extracting modal decomposition. In POD, mode 1 usually contains the mean field and the higher modes contain the fluctuations of the field (Berkooz et al. 1993). Using POD, a variable $A(\phi ,\varepsilon )$ can be written as (Berkooz et al. 1993; Rönnberg and Duwig 2021):

4 $$\begin{aligned} A(\phi ,\varepsilon ) = \sum _{i=1}^{\infty } a_i(\varepsilon )\Xi _i(\phi ), \end{aligned}$$

where *i* is the mode number, $\Xi _i$ and $a_i(\varepsilon )$ are the mode and the coefficient associated with the mode *i*, respectively. This decomposition is achieved by using Singular Value Decomposition (SVD). In SVD we decompose the matrix $A_{N\times m}$ into (Chatterjee 2000):

5 $$\begin{aligned} A = U\Sigma V^T, \end{aligned}$$

where *U* and *V* are orthogonal matrices of dimension $N\times N$ and $m \times m$, respectively, and $\Sigma$ is a diagonal matrix of dimension $N \times m$. The diagonal elements of $\Sigma$, called singular values, contains non-negative numbers $\sigma _i$ ($i=1,2,..,r;\, r=min(N,m)$), which are arranged in decreasing order ($\sigma _1 \ge \sigma _2 \ge \sigma _3 \cdots \ge \sigma _r$). Singular values are unique to the matrix *A* and are equal to the eigenvalues of the matrix *A*. It is worth noting that Eqs. 4 and 5 are equivalent to each other. This is achieved by taking $U\Sigma =Q$, giving:

6 $$\begin{aligned} A = QV^T = \sum _{k=1}^{m} q_k v_k^T, \end{aligned}$$

where *k* is the column number of matrix *Q* and *V*. In Eq. 6, $q_k$ corresponds to $a_i(\varepsilon )$ and $v_k^T$ corresponds to $\Xi _i(\varepsilon )$. A rank *r* approximation to matrix *A* can be obtained by replacing matrix *U* and *V* with the matrices of their first *r* columns and taking only the first *r* rows and columns of $\Sigma$. The mode *r* of *A* is obtained by removing the rank *r* approximation of *A* from the rank $r+1$ approximation of *A*.

**Fig. 5:**
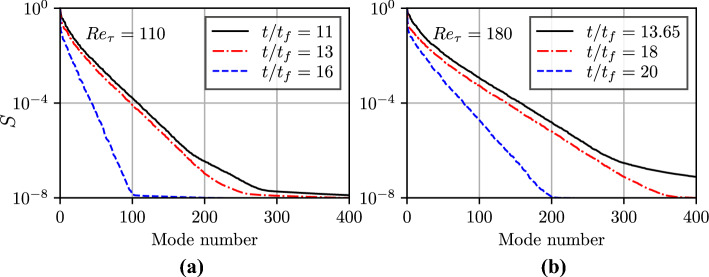
Variation of relative information content, *S*, with the number of POD modes, *m*, at different time instance for the wall heat flux $\Phi _w$ for $Re_\tau =$ **a** 110 and **b** 180

In the present work, the instantaneous wall heat flux and wall shear stress matrix are decomposed using POD into $a_i(x,t)$ and $\Xi _i(z,t)$. The temporal evolutions of the correlation coefficient of different POD modes of wall heat flux with wall shear stress, $r_{\Phi m\tau }$, during the HOQ event are exemplified in Figs. 4a and b for $Re_\tau =110$ and 180, respectively. The total number of samples for each plane for each snapshot corresponds to $1920 \times 720 \, (3200 \times 1200)$ grid points for $Re_\tau =110 \, (180)$ cases and the statistical convergence is ensured by establishing no significant differences ($\sim 0.1\%$) as a result of halving the sample size. The correlation coefficient between the $i^{th}$ mode of heat flux and shear stress $r_{\Phi m \tau }$ at different time instant is presented in the Figs. 4a and b. The correlation coefficients of the $i^{th}$ mode of shear stress and heat flux $r_{\tau m \Phi }$ at different time instances were also evaluated and found to be equal to $r_{\Phi m \tau }$. Figures 4a and b show that the negative correlation between the first POD mode of $\Phi _w$ is principally responsible for the negative correlation and this correlation weakens with the progress of HOQ. The correlation between the first POD mode of $\tau _w$ and $\Phi _w$ is qualitatively similar to those shown in Figs. 4a and b, and thus are not explicitly shown here. Hence, the first modes of $\Phi _w$ and $\tau _w$ play a key role in determining their respective statistical behaviours. This can be substantiated by the variation of the singular values of different modes normalised by the largest singular value (i.e., the singular value associated with mode 1) *S* (which is often referred to as the relative information content) with the number of POD modes *m* for the wall heat flux $\Phi _w$ in Figs. 5a and b for $Re_\tau =110$ and 180, respectively. It can be seen from Figs. 5a and b that the number of modes for which the relative information content *S* assumes non-negligible values decreases with the progress of HOQ. It has been shown elsewhere (Ahmed et al. 2023) that the turbulence kinetic energy within the boundary layer decreases with the progress of HOQ in this configuration and this decay in turbulence implies a smaller number of modes with significant *S* values at later times. A comparison between Figs. 5a and b also reveals that the range of modes for which *S* assumes non-negligible values also increases with an increase in $Re_\tau$.

**Fig. 6:**
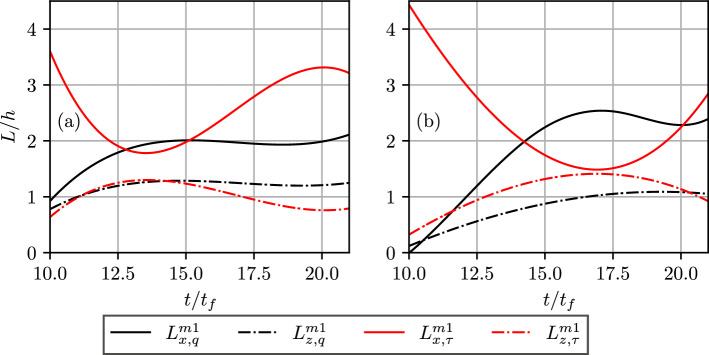
Temporal evolutions of the integral length scales based on the first POD modes of wall heat flux (i.e., $L_{x,q}^{m1}$ and $L_{z,q}^{m1}$) and wall shear stress (i.e., $L_{x,\tau }^{m1}$ and $L_{z,\tau }^{m1}$ for $Re_\tau =$ **a** 110 and **b** 180 cases

**Fig. 7:**
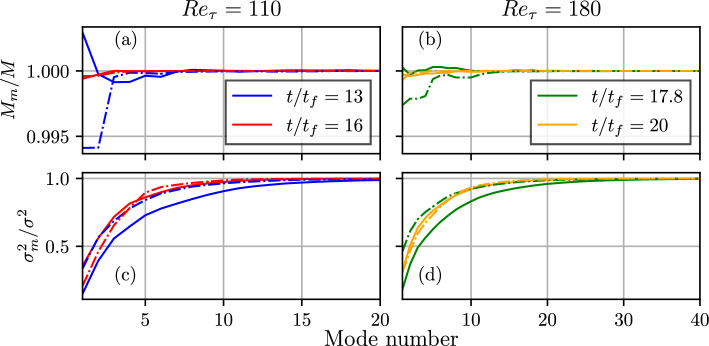
Fractions of the cumulative contribution of the first *m* nodes to the mean, $M_m/M$, **a**,**b** and variance, $\sigma _m^2/\sigma ^2$, **c**,**d** of $\Phi _w$ (solid line) and $\tau _w$ (broken line) at different time instants during HOQ for $Re_\tau =110$ (a,c) and 180 (c,d) cases

The temporal evolutions of the integral length scales based on the first POD modes of wall heat flux magnitude (i.e., $L_{x,q}^{m1}$ and $L_{z,q}^{m1}$) and wall shear stress (i.e., $L_{x,\tau }^{m1}$ and $L_{z,\tau }^{m1}$) are shown in Fig. 6, which shows qualitatively similar behaviours as those of $L_{x,q}, L_{z,q}, L_{x,\tau }$ and $L_{z,\tau }$, as shown in Figs. 2e and f. Although the temporal variations of $L_{x,q}^{m1}, L_{z,q}^{m1}, L_{x,\tau }^{m1}$ and $L_{z,\tau }^{m1}$ are mostly similar to $L_{x,q}, L_{z,q}, L_{x,\tau }$ and $L_{z,\tau }$, the ratio between mode 1 length scale and the original data is $~\mathcal {O}(10)$. Since the first mode captures only the most energetic mode, more number of modes need to be added to accurately depict the spatial variation of the heat flux magnitude and wall shear stress, and thus retrieve the actual length scale. As mentioned previously, the POD modes for homogeneous data are equivalent to the Fourier modes of the two-point correlation function of the data (Berkooz et al. 1993). In LES, the small scales are not completely resolved, and sub-grid closures are used to model these scales. This implies that the data generated for wall heat flux and wall shear stress from LES would mimic the data generated by the first few POD modes of the DNS data. This implies that Large Eddy Simulations (LES) and experimental measurements with appropriate resolutions to capture the first POD modes of wall heat flux magnitude and wall shear stress could be used to obtain at least the qualitative nature of the integral length scale variations. However, just the first mode of POD is not sufficient to capture the mean and variances of the wall heat flux and wall shear stress. The fractions of the cumulative contribution of the first *m* modes to the mean, $M_m/M$, and variances, $\sigma _m^2/\sigma ^2$, of $\Phi _w$ and $\tau _w$ are shown in Fig. 7. Here $M_m$ is the mean of the rank *m* approximation of $\Phi _w$ or $\tau _w$, *M* is the mean of $\Phi _w$ or $\tau _w$, as appropriate, $\sigma _m^2$ is the variance of rank *m* approximation to $\Phi _w$ (calculated as $\langle (\Phi _{w,m}-\bar{\Phi }_{w,m})^2\rangle$, where $\Phi _{w,m}$ is the rank *m* approximation to $\Phi _w$) or $\tau _w$, as appropriate, and $\sigma ^2$ is the variance of $\Phi _w$ or $\tau _w$. Figures 7a–d demonstrate that at least 10 modes of POD (i.e., $m\ge 10$) are necessary to capture the mean and variance of both $\Phi _w$ and $\tau _w$ with an almost 100% accuracy. Moreover, it can be seen from Figs. 7a–d that the number of POD modes to obtain the mean and variance of both $\Phi _w$ and $\tau _w$ with an almost 100% accuracy decreases with decreasing $Re_\tau$ and also the progress of HOQ for a given value of $Re_\tau$, which is consistent with the relative information content variations shown earlier in Fig. 5.

## Conclusion

The interdependence of wall heat flux and wall shear stress during HOQ of statistically planar turbulent premixed flames within turbulent boundary layers representative of $Re_\tau =110$ and 180 are analysed using three-dimensional DNS data. It has been found that the temporal evolutions of mean wall heat flux and wall shear stress are considerably different. In all cases, the mean wall heat flux magnitude increases with time as the flame approaches the wall and eventually assumes a maximum value before decreasing with time with the progress of flame quenching. By contrast, the mean wall shear stress decreases during FWI before settling to a constant value, which is smaller than the wall shear stress in the unburned gas. The integral length scales in the streamwise and spanwise directions for wall heat flux are found to be similar to the corresponding length scales of the wall shear stress during FWI. The integral length scales of wall heat flux in both streamwise and spanwise directions have been found to grow with time after the maximum mean heat flux magnitude is obtained for both $Re_\tau$ cases considered in this work. However, the integral length scale of wall shear stress in the streamwise (spanwise) direction decreases (increases) with time before reaching the minimum (maximum) value, which is followed by an increasing (decreasing) trend with time.

In this work it has been shown that the correlation coefficient between the wall heat flux magnitude and wall shear stress becomes increasingly negative as the mean wall heat flux increases with time but the correlation weakens as the mean heat flux decreases with time with the progress of flame quenching. The redistribution of momentum as a result of thermal expansion is responsible for the decrease in the mean wall shear stress and the negative correlation between the wall shear stress and the wall heat flux magnitude. Note that the negative correlation between the wall shear stress and wall heat flux through the process of thermal expansion, momentum redistribution, and the wall heat flux is important to consider from a modelling viewpoint. This will help in designing the cooling load and identifying materials (eg. coatings) for combustor walls for industrial applications. In this work it has been demonstrated that the first (i.e., most energetic) POD modes of wall shear stress and the wall heat flux magnitude are sufficient to capture the qualitative nature of the correlation between these quantities and their spatial variations. Therefore, LES simulations and experimental measurements which can resolve the first POD modes of wall shear stress and the wall heat flux magnitude are at least capable of providing the correct qualitative behaviour regarding the integral length scales of the spatial variations of wall heat flux and wall shear stress and the correlation between these quantities. However, typically 10-20 most energetic POD modes are needed to accurately capture the mean and variance of wall heat flux and wall shear stress. The number of modes needed to capture the mean and variance of these quantities increases with increasing $Re_\tau$, but decreases with the progress of HOQ due to the weakening of turbulence effects.

## Data Availability

No datasets were generated or analysed during the current study.
